# The method to identify invasive mechanical ventilation with Japanese claim data

**DOI:** 10.1186/s40560-024-00760-0

**Published:** 2024-11-12

**Authors:** Ayaka Sakamoto, Yoshiaki Inoue

**Affiliations:** 1 Department of Emergency and Critical Care Medicine, University of Tsukuba Hospital, 2-1-1 Amakubo, Tsukuba, Ibaraki 305-8576 Japan; 2 Graduate School of Comprehensive Human Sciences, University of Tsukuba, 1-1-1 Tenno-Dai, Tsukuba, Ibaraki 305-8577 Japan; 3 Department of Emergency and Critical Care Medicine, Institute of Medicine, University of Tsukuba, 1-1-1 Tennodai, Tsukuba, Ibaraki 305-8577 Japan

**Keywords:** Invasive mechanical ventilation, Claim data, Procedure code, Japan

## Abstract

Dr. Ohbe et al. reported that only 40.4% of patients who underwent invasive mechanical ventilation were treated in intensive care units, with significant variations in intensive care unit admission rates observed between hospitals and regions using Japanese claims data. The issue of validation when using claim data has been reported in previous studies. The definition of invasive mechanical ventilation used by Dr. Ohbe et al. appears overly broad, encompassing non-invasive mechanical ventilations via nasal mask and manual ventilation. We discuss the limitation of their method in identifying invasive mechanical ventilation, which is critical for defining the study population.

**To the Editor,**

We read with great interest the study by Dr. Ohbe et al. This retrospective cohort study, based on the nationwide Japanese inpatient administrative database, found that only 40.4% of patients who underwent invasive mechanical ventilation (IMV) were treated in Intensive Care Units (ICU), with significant variation in ICU admission rates across hospitals and regions [1]. However, we have concerns regarding the method used to identify the target population underwent IMV.

The limitations of claims data have been discussed in previous studies. The concordance between diagnostic codes in claim data and actual diagnoses often varies by disease [2], and the incidence and mortality rates estimated using claim data may differ from actual situations [3]. Therefore, efforts are commonly made to combine multiple codes to accurately identify target populations [4]. Different methods have been employed in prior studies using claim data to identify the same condition, and the positive predictive value (PPV) of these methods has varied [4]. Since sensitivity and PPV have a trade-off relationship [5], it is crucial to carefully select the most appropriate codes based on the specific research objectives.

In Dr. Ohbe et al.’s study, only the Japanese procedure code for “artificial ventilation (J045)” was used to detect IMV. This code is too broad to accurately identify IMV, as it includes a wide range of interventions, such as manual ventilation (e.g., bag-valve-mask ventilation) and non-IMV via nasal mask in case of acute respiratory failure (P_a_O_2_/F_i_O_2_ ≤ 300 or P_a_CO_2_ ≥ 45 mmHg). While home mechanical ventilation or ventilation on the same day as general anesthesia is excluded, artificial ventilation during CPR can be claimed by J045, which contradicts Dr. Ohbe et al.’s explanation. To grasp the situation of IMV appropriately and to conduct future research, it is desirable that the definition of IMV using procedure code will be reconsidered.

The J044 procedure code for “lifesaving endotracheal intubation” may improve the PPV for detecting IMV, though it may reduce sensitivity. Intubation for general anesthesia or diagnostic exam cannot be claimed by J044, therefore we could not include the patients who received IMV following these procedures with intubation. In addition, there is no procedure code for extubation, which makes it difficult to detect transition from IMV to non-IMV using claim data.

Identifying IMV using procedure code is complex. Therefore, selecting an appropriate combination of procedure codes based on the study objective is essential. Using only the code for “artificial ventilation (J045)” may be too broad a definition to accurately detect IMV, although some prior studies on IMV have also relied solely on it [6, 7]. The most critical factor is understanding the applicable conditions for each procedure code, and it is necessary to consult with doctors or other medical staffs familiar with clinical practice in that area. Following these clarifications, a validation study is warranted to confirm the accuracy and reliability of the procedure codes for identifying IMV in future research.

For future research utilizing big data from insurance claims, careful attention must be paid to the coding methods used.

## Data Availability

Not applicable.

## Abbreviations

IMV: Invasive mechanical ventilation
ICU: Intensive care unit
PPV: Positive predictive value
CPR: Cardiopulmonary resuscitation
